# Supplementary material for: Patterns of Intron Gain and Loss in Fungi
Source: PLoS Biol. 2004 Nov 30;2(12):e422. doi: 10.1371/journal.pbio.0020422 (PMC532390; doi:10.1371/journal.pbio.0020422)
Supplement: Table S1 — Also available at http://genes.mit.edu/NielsenEtAl/. (4.3 MB ZIP). [file pbio.0020422.st001.zip › NielsenEtAl/html/227.html]

AN0568.1.NCU00289.1.MG08917.1.FG04683.1


```
 CLUSTAL W (1.82) Multiple Sequence Alignments - Introns Inserted


Sequence 1: NCU00289.1	659 aa
Sequence 2: MG08917.1	648 aa
Sequence 3: FG04683.1	559 aa
Sequence 4: AN0568.1	581 aa
Alignment Length: 675 aa
Number Identitical Residues: 101 aa
Alignment Score (without introns) 7199


MG08917.1 	MDVFDPSVARLLATTSGQQSTSPLESEAEVGLQSGTGEGPEASLSGESSTSQGGAPSVPA
NCU00289.1	MEPFSPEAPHCNGTSS---SSSPAVSSDSPGTAT-SGTVRNGSVATTTSQPSVPPPSVPA
FG04683.1 	-------------------------MLPPIELWTGSGTRRN-------------ITSVPT
AN0568.1  	------------------------MNEHYLPAKALTQQPRATG-------------PSLL
          	                                 : :      .             .   

MG08917.1 	ACLAC0RGKHLKCDGATP-CSRCVAS--ASECVYIASRRGYKGPRKS----NVAHNPNKR
NCU00289.1	ACLPC0RTKHLRCDGNHP-CGRCTTTGIVADCAYIASRRGYKGPRRNPNNPTPGANPNKR
FG04683.1 	ACLVC0RRRHLKCDGQCP-CSRCITAN--LDCAYVASRRGYNGRKRMSR--AAAEKPFLI
AN0568.1  	ACLLC~RHKHLKCDGATPVCSRCAATG--AECQYTPSRRGYKG-------------PSKK
          	*** * * :**:***  * *.** ::.   :* * .*****:*             *   

MG08917.1 	FASSPTPGSAPDDDSCPMLLGASP-SGSTVTTGSYT-QAPVLS-----QFVAPPVGANMQ
NCU00289.1	HASSSPPYAGTTPDSCPMMLGHAPVTMATPSVHAFN-PSVVLPDQSQLSFPSTPSVASIP
FG04683.1 	AASTAITIDSAPPVAGLEFTSHLDPFMFSGILDSLSGVNTTLPVG---TSCQTAASTTLP
AN0568.1  	RRANPSSPEQLPADLAPSFDPNVGFYNVPVDWNALN-PYPYVPSA---TLPASTSSGSSP
          	  :.. .           :         .    : .     :.         ..   .  

MG08917.1 	LYRNPFLMAGGGSSSSSANTNTNHAGSSHMRSQLSMPERCLTSFYQNFHASHPCVLPRDA
NCU00289.1	VYRNNYMMDG------TAMTLATQAAPPIQPPAASLPERCFEAFYHFFHAGHPFVLPKRN
FG04683.1 	VLSLQNPLGG--------IDHPNPGFGFEVPRALPQRERYIDAFYQKFFAAHPFILPKEP
AN0568.1  	QFTEQPGASQ-------QVVTKNAPLTPESSSSLSNDGYLVDIYYQFFHPSHPILPPIKT
          	                      .           .     .  :*: *...** : *   

MG08917.1 	LERVIREGP-AKLEHLLAAMRYLGSLYLDAGPARAQYFDEAVSLAYHPGCPRDGFLAQTL
NCU00289.1	LVELLKSNSEPNLPVLMAAMRYIGSLYVDAGPAKARFLDEAIQLCYAPGVRKDGFLIQAL
FG04683.1 	LLSLAQGTS---VEPLITAIRWIGSLYIDK-HASQSLLKDAFRLIDG-NHLREGFLVQAR
AN0568.1  	LYHNR------VPPYLEQVIKFVGSHFTPAASSETYRPGIMTTVMEQEGTLEK---IQAL
          	*              *  .::::** :   . :          :    .  ..    *: 

MG08917.1 	LLLSIGQDGSCNQARANQFMADAERLAVEIGLNTRQFATANGRGSSVVEESWRRTWWELY
NCU00289.1	LLIIIGLDGSCEQERARELLAICERYAIEIDLNKREFAVMHGRGDPIMEESWRRTWWDLY
FG04683.1 	LLLIIGLDGIRQRKKANKLMSEARDISVQIGMNTSSFATTHGQGIPILEESWRRTWWELY
AN0568.1  	LLLAIVLHSRNERDKAKDCLITAVDLAFELGLHTRDFATTMGGGNPLREECLRRTWWELF
          	**: *  ..  :: :*.. :  .   :.::.::. .**.  * * .: **. *****:*:

MG08917.1 	VVNGMIAGVHRITNFALYDVAADVGLPCEEHQFLSG0N-IPMPMYMEDFDDQLFSGEDRE
NCU00289.1	VCDGMIAGVHRVTNFLLFDIMTDVGLPCEEEEYHKG0C-IPSPMSLEDFDDKEFQDEDRE
FG04683.1 	VVDALMSGVHLTNTFALYEVPTDVGPPCEENQYRSG0Q-IPPCMHLNDTENIGLFD-GKP
AN0568.1  	IIEAMLTALGLQKSPRTHHVPLEVPLPCEERIYQDG~IEPPPPPTIAQFDERVFADEERD
          	: :.:::.:   ..   ..:  :*  ****. : .*  . *    : : ::  : .. : 

MG08917.1 	FSSFAYRIASARNLGRMMRTPPMLSPDDDNLAKLEALLTNWKMHLPRSRQDALNKKCQLD
NCU00289.1	FSSFAYRVAAARNLGRMMRMPNVMFPDAAEVDKMEAHLSNWRMHLPDNKRDDLDKNCQLD
FG04683.1 	FSSYTYRIQCACILGRLQRMP-------TNVDYIDKLLANWMLRLPASKYAAFYDG-ALD
AN0568.1  	FSSYTYRIEAARILGRVVAIQDLVEGQQDQVESIDARITSFFHHLPESKAELLRPDGTVD
          	***::**: .*  ***:         .  ::  ::  ::.:  :** .:   :     :*

MG08917.1 	EMMFQALFINHA2CTIMLHQPHSQLDSSPSRSVTSCAPHMPVSSGDAFN--THTRHTVTA
NCU00289.1	EMMFQAHFITHA2CTIMLHQPLSQLDSSPVQAVNSCAPHRPVHSGDQYN--AHTRHTVTA
FG04683.1 	EMMFQAIMMWHA~ISILLHQPHSQLDPSPTYHIKACAPNTPAMSSDEFN--PHTKRTIRA
AN0568.1  	EMMFQATMVVNG~TAIYLHFPRSDLLSSPAVAAEVICGHHGPCSVPAFSHHSHAMKALKA
          	****** :: :.  :* ** * *:* .**       . :    *   :.  .*: ::: *

MG08917.1 	ACEVSRMVTHAVPLLSHTHFFTCVLTLSSIVHLNKWALYFLQ-DEDDLRQQIRLNIGALQ
NCU00289.1	ACEISKMITQAVPVTSHTHFFTCVVTLSSIVHLSKWALYMIE-DEADLRQQIRLNIGALN
FG04683.1 	AEELSKLITHRVTLISHTHFFAYMVTLSSTIHLSKWSLAFVAPDDEEIRQHMRQSIGALI
AN0568.1  	ASEISSLASIRMPVVKHTPFFICSLVMSSIVQLAACSVKAGQ-MPDPSRDRLALTIGVFK
          	* *:* : :  :.: .** **   :.:** ::*   ::          *:::  .**.: 

MG08917.1 	RISAVWQNAAKAGGQVRGVAQEIYRAKKAQQISPAFWVAFTQEQMLTSLNADEGIMNDFD
NCU00289.1	KLSKVWKAANTAWTQVKGVAQEIYRQKKVQQITPAFWVGYTQEQMMTSIQADESIISEFD
FG04683.1 	KYAEKWPIAQHLGCQVKDIAKEVYMMKKRQ----------SQE-----------------
AN0568.1  	TLANTWAISQSIMRQIKAVARDVMDMGLRP--------TMAMDQIDLNTVLDNNGRFWLA
          	  :  *  :     *:: :*:::                 : :.   .   ...      

MG08917.1 	SILPPQVTRP--------
NCU00289.1	QGLIAQVGIAQVGVTQAQ
FG04683.1 	------------------
AN0568.1  	EALPR-------------
          	.
```
